# Phenotypic Profiling and Activation-Associated Expression of CD99 Ligands on Human Leukocytes

**DOI:** 10.3390/biology15010086

**Published:** 2025-12-31

**Authors:** Myint Myat Thu, Nuchjira Takheaw, Witida Laopajon, Watchara Kasinrerk, Supansa Pata

**Affiliations:** 1 Division of Clinical Immunology, Department of Medical Technology, Faculty of Associated Medical Sciences, Chiang Mai University, Chiang Mai 50200, Thailand; myintmyatthu_myint@cmu.ac.th (M.M.T.); nuchjira.t@cmu.ac.th (N.T.); witida.l@cmu.ac.th (W.L.); watchara.k@cmu.ac.th (W.K.); 2 Biomedical Technology Research Center, National Center for Genetic Engineering and Biotechnology, National Science and Technology Development Agency at Faculty of Associated Medical Sciences, Chiang Mai University, Chiang Mai 50200, Thailand

**Keywords:** recombinant CD99 human IgG fusion protein, CD99 ligands, activation markers, immunophenotyping, immune regulation

## Abstract

The immune system comprises a complex network of cells that continuously change during activation, infection, and the maintenance of balance. Immunophenotyping offers valuable insights into the regulation of immune responses. We systematically characterized the expression profile of CD99 ligands across distinct immune cell subsets using both conventional and high-dimensional flow cytometry. CD99 ligands were detected on NK cells and monocytes under both resting and IL-2-activated conditions, with non-classical monocytes and CD56 Dim NK cells exhibiting the highest expression levels. Notably, ligand expression in these subsets was further enhanced following IL-2 activation. In contrast, T lymphocytes (CD3^+^) displayed low basal levels of CD99 ligand expression, which increased modestly upon activation. Cellular activation was accompanied by an expansion of specific immune phenotypes characterized by elevated CD99 ligand expression alongside the upregulation of activation markers such as CD69 and CD137. Collectively, these findings suggest that the expression of the CD99 ligands may serve as an indicator of immune activation and demonstrate subset-specific regulation, particularly in response to IL-2 stimulation. These findings have revealed the distinct expression patterns of CD99 ligands, emphasizing their crucial role in modulating immune responses.

## Simple Summary

Protein–ligand interactions are ubiquitous in biological cells. Transmembrane protein CD99 is expressed on all leukocytes, but information on the distribution and behavior of its ligands is still lacking. Here, we report the distribution of CD99 ligands across various immune cell types and examine how their levels vary upon immune cell activation. Our findings indicate that CD99 ligands are predominantly expressed on natural killer (NK) cells and monocytes, with their expression further increasing after interleukin (IL)-2 stimulation. These data suggest that CD99 expression is associated with immune activation and may serve as a marker for activated immune cells.

## 1. Introduction

CD99, a transmembrane protein expressed broadly on both hematopoietic and non-hematopoietic cells, has been shown to play an essential role in various biological processes [1,2]. In a recent study, we have demonstrated that high-quality recombinant CD99 proteins with active biological activity can be produced under optimized conditions [3]. Moreover, the existence of CD99 ligands has been revealed, and several potential ligand candidates have been identified using LC-MS/MS [4]. Growth and differentiation factor 6 (GDF6) in Ewing sarcoma, paired immunoglobulin-like type 2 receptors (PILRs), CD99 antigen-like protein 2 (CD99L2), and CD99 itself were shown to act as ligands of CD99 and contribute to the regulation of the immune system [5,6,7]. Our previous work also showed that the interaction between CD99 and its ligands is involved in regulating cytokine production [8]. These findings indicate that CD99 plays a role in immunoregulation. However, the distribution of CD99 ligands across immune cells is still unexplored. Understanding how these ligands are distributed will guide future functional studies and potential therapeutic strategies. Previously, we demonstrated the presence of CD99 ligands on NK cells, monocytes, and dendritic cells [8], but there is no information on whether CD99 ligands are expressed on activated populations of leukocytes. The exact expression pattern, in relation to the cytokine activation state, remains undefined.

In this study, we further examined the expression of CD99 ligands on IL-2-activated PBMC populations. High-dimensional analysis can reveal complex phenotypic patterns that are often missed by conventional approaches. We investigated the distribution of CD99 ligands among various phenotypes of unactivated and IL-2-activated PBMC populations, including NK cells, CD3^+^ cells, and monocytes, using unsupervised hierarchical clustering algorithms, UMAP, FlowSOM, and Cluster Explorer. The conventional analysis showed that CD99 ligands were expressed on unactivated and IL-2-activated NK cells and monocytes but not on the CD3^+^ population. The expression levels were higher in the activated condition compared to the unactivated condition. High-dimensional analysis showed that activation causes different phenotypes to vary in CD99 ligand expression. The upregulation of ligands correlates with an increased number of cells expressing activating markers. Activation facilitates the clustering of ligands on specific phenotypes, thereby elevating CD99 ligand levels in comparison to unactivated states.

## 2. Materials and Methods

Detail information of reagents used in this study are described in Table S2.

### 2.1. Antibodies

PECF594-conjugated anti-CD3 was purchased from BD Biosciences (San Jose, CA, USA). PECy5-conjugated anti-CD56, FITC-conjugated anti-CD14, Brilliant violet (BV)711-conjugated anti-CD16, BV421-conjugated anti-CD69, PECy7-conjugated anti-CD336, and PECy7-conjugated anti-CD137 mAbs were purchased from BioLegend (San Diego, CA, USA). The recombinant protein CD147Rgwas produced in our laboratory [9].

### 2.2. PBMC Isolation

Peripheral blood mononuclear cells (PBMCs) were isolated from healthy donors using Ficoll–Hypaque (IsoPrep) (Robins Scientific Corporation, Sunnyvale, CA, USA) gradient centrifugation. Heparinized whole blood was mixed with phosphate-buffered saline (PBS) at a 1:1 ratio. This diluted blood was overlaid onto Ficoll–Hypaque solution and then spun at 400× *g*, 25 °C for 30 min with a break-off setting. After centrifugation, the PBMCs were harvested from the white ring at the interphase of Ficoll–Hypaque and the plasma layer. The isolated PBMCs were maintained in RPMI-1640 medium supplemented with 10% fetal bovine serum (FBS; Gibco, Grand Island, NY, USA), 40 mg/mL gentamicin, and 2.5 mg/mL amphotericin B (10% FBS-RPMI 1640). For activation, the isolated PBMCs were incubated with 250 IU/mL of IL-2 (Immunotools, Friesoythe, Germany) for 24 h and cultured at 37 °C in a 5% CO_2_ incubator.

### 2.3. Determination of CD99 Ligands by Immunofluorescence Staining with DTSSP

Previously produced recombinant CD99HIgG and CD147Rg proteins were labeled with EZ-Link Sulfo-NHS-LC-Biotin (Thermoscientific, Rockford, IL, USA) as described elsewhere [8]. To determine the cellular distribution of CD99 ligands on unactivated and IL-2-activated PBMC populations, PBMCs were prepared by Ficoll–Hypaque density gradient centrifugation. The cell concentration was adjusted to 2 × 10^6^ cells/mL. The isolated PBMCs were treated with or without 250 IU/mL IL-2 and incubated at 37 °C in a 5% CO_2_ incubator overnight. PBMCs were then harvested and suspended in a concentration of 2 × 10^7^ cell/mL in a blocking solution (Human TruStain FcX^TM^) (BioLegend, San Diego, CA, USA) to block Fc receptors. PBMCs in the blocking solution were rotated at 4 °C for 30 min. After that, Fc-blocked PBMCs were stained with 40 ug/mL of biotinylated CD99HIgG or protein control, CD147Rg, for 1 h on ice, followed by adding 2mM crosslinker DTSSP (3,3′-dithiobis sulfosuccinimidylpropionate) to covalently link between recombinant CD99 and its ligands on PBMC and to stabilize the weak or transient protein–ligand interactions. After incubating on ice for 2 h, the reaction was stopped with 20mM Glycine in PBS and washed.

The bound biotinylated proteins were detected with Streptavidin-PE (Strep PE). The surface marker membrane proteins were stained with PE/Dazzle-anti-CD3 mAb, FITC-anti-CD14 mAb, and PE/Cy5-anti-CD56 mAb for determination of NK cells, CD3^+^ cells, and monocytes. The distribution of CD99 ligands on different phenotypes was examined by staining with BV711-anti-CD16 mAb, PECy7-anti-CD336 mAb, BV421-anti-CD69 mAb, and PECy7-anti-CD137 mAb, respectively. The ligand expression was analyzed using flow cytometry (BD FACSCelesta^TM^) (BD Biosciences, San Jose, CA, USA) and FlowJo v.10.10.0 using conventional and high-dimensional analysis. FlowJo plugins software (UMAP, FlowSOM and Cluster Explorer) were used for high-dimensional analysis. Dimensionality reduction analysis was performed using UMAP (v4.0.4), and phenotypically distinct cell clusters on the resulting UMAP plot were identified by FlowSOM (v4.0.0). Cluster Explorer in FlowJo v.10.10.0 was used after completing dimensionality reduction and population identification through clustering and overlaid dimensionality reduction plots, profile charts, and heatmaps were generated. Median fluorescence intensity (MFI) values of surface markers and CD99 ligands were exported from the heatmap of Cluster Explorer.

### 2.4. Statistical Analysis

Statistical analysis was carried out with GraphPad Prism 10 software (San Diego, CA, USA). Significant values were analyzed using paired and unpaired t tests, and *p* values ≤ 0.05 were considered significant.

## 3. Results

### 3.1. Expression of CD99 Ligands on NK Cells, Monocytes, and CD3^+^ Cells Determined by Conventional Analysis

**Figure 1:**
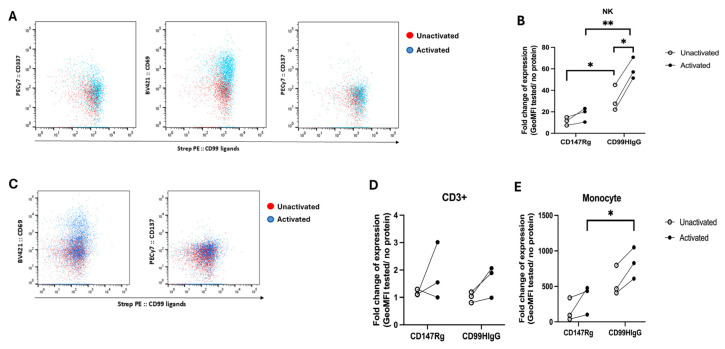
Expression of CD99 ligands on unactivated and IL-2-activated NK cells, CD3^+^ cells, and monocytes. (**A**) The representative overlayed dot plot for the expression of NK cell-activating receptors in unactivated and IL-2-activated NK cells. The red dot plot represents the unactivated condition, while the blue dot plot indicates the activated condition. The Y axis shows the expression of NK cell activation markers, CD69, NKp44, and CD137, and the X axis shows the expression of CD99 ligands. (**B**) The graph indicates the fold change in geometric mean fluorescence intensity (GeoMFI) between conditions with and without recombinant protein. The ligand expression on NK cells, both with and without activation with IL-2 (n = 3), is shown. CD147Rg, the recombinant CD147 protein, was used as a control. Each dot represents an individual subject, and the horizontal lines connect the corresponding subjects between unactivated and activated conditions. Statistical analysis was performed with an unpaired *t*-test. * *p* ≤ 0.05, ** *p* ≤ 0.01. (**C**) The representative overlayed dot plot for the expression of activating receptors in unactivated and IL-2-activated CD3^+^ cells. The red dot plot represents the unactivated condition, while the blue dot plot indicates the activated condition. The Y axis shows an expression of CD3^+^ cell activation markers, CD69, or CD137, and the X axis shows an expression of CD99 ligands. The graph illustrates the fold change in GeoMFI between conditions with and without recombinant protein. (**D**) The CD99 ligand expression with or without IL-2 on CD3^+^ cells (n = 3) and (**E**) on monocytes (n = 3) is analyzed. Each dot represents each subject, and a horizontal line connects each corresponding subject. Statistical analysis was performed using an unpaired *t*-test. * *p* ≤ 0.05.

The cellular distribution of CD99 ligands was investigated using DTSSP to covalently link CD99HIgG with its ligands expressed on both unactivated and IL-2-activated NK cells. CD3^−^ CD14 cells were first gated, and then the population of CD3^−^ CD56^+^ was identified as NK cells (gating strategy is shown in Supplementary Figure S1). The frequency of CD69-expressing cells was markedly increased on IL-2-activated PBMCs compared to the unactivated condition. In addition, the expression of NKp44 and CD137 was slightly higher (Figure 1A). The results indicated that NK cells are successfully activated after being treated with IL-2. CD99 ligands were expressed in both unactivated and activated NK cells (Figure 1B). However, the expression level of CD99 ligands was significantly higher in the condition treated with IL-2 compared to the untreated condition (Figure 1B). Compared with non-irrelevant control CD147Rg, a soluble recombinant fusion protein consisting of the extracellular domain of the human CD147 protein fused to the hinge and Fc region of human IgG1, the CD99 ligand expression levels were markedly higher in both unactivated and activated conditions. These data suggest that the expression was specifically due to the interaction between CD99 and its ligands.

To explore the expression of CD99 ligands on monocyte and CD3^+^ populations, cells were gated according to Supplementary Figure S2. As shown in Figure 1C, CD3^+^ cells were successfully activated with the increased expression of activation markers CD69 and CD137 on the IL-2-activated population. When examining CD99 ligand expression levels on the CD3^+^ population, no significant difference was observed compared to the CD147Rg control in both unactivated and IL-2-activated conditions. However, the expression level of CD99 ligands tended to increase in IL-2-activated conditions compared to unactivated conditions in all three donors tested, although it was not statistically significant (Figure 1D).

As expected, CD99 ligands were expressed on monocytes in both unactivated and IL-2-activated conditions (Figure 1E). Importantly, the expression level of CD99 ligands in IL-2-activated monocytes demonstrated a significant difference between CD99HIgG and the CD147Rg control (Figure 1E).

### 3.2. High-Dimensional Analysis of Distribution of CD99 Ligands on PBMCs Before and After Activation with IL-2

**Figure 2:**
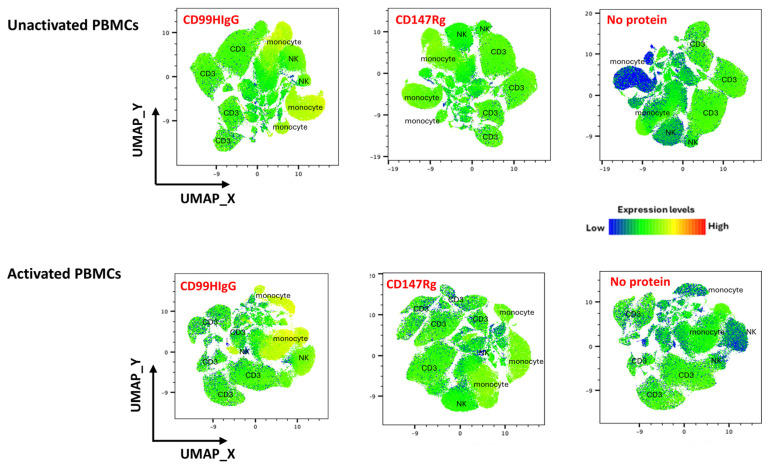
UMAP-based dimensionality reduction showing CD99 ligand expression on PBMCs before and after activation with IL-2. A representative UMAP plot illustrates CD99 ligand expression compared with the control conditions, CD147Rg and no protein. UMAP plot revealed three major populations (CD3^+^, NK, and monocyte) based on marker expression. The color intensity in UMAP indicates the expression level of ligands within each identified population, ranging from low (blue) to high (red) colors.

Our results show that CD99 ligands were expressed on the surface of PBMC populations, including NK cells, CD3^+^ cells, and monocytes, both before and after activation with IL-2. We further used UMAP-based dimensionality reduction, a type of high-dimensional analysis, to discover the cellular distribution of CD99 ligands on unactivated and IL-2-activated PBMCs. A representative heatmap of UMAP is shown in Figure 2. Compared to the controls, CD147Rg and no protein, CD99HIgG staining showed higher expression levels (yellow color) in PBMCs, particularly in activated ones, with the strongest expression observed on monocytes. This data corresponds to the findings of conventional flow cytometry analysis.

To further examine the distribution of CD99 ligands across different phenotypes of NK cells, CD3^+^ cells, and monocytes, we applied the unsupervised hierarchical clustering algorithms FlowSOM and Cluster Explorer. First, CD3^+^ and CD3^−^ populations were gated using 2D analysis, and the CD3^−^ population was further analyzed by UMAP to identify monocytes and NK cells (Figure S3). The heatmaps of CD56, CD16, CD14, and NKp44 expression levels are shown in Figure S3B. Based on the CD56 and NKp44 expression levels defined by UMAP, two NK populations (NK1 and NK2) and non-NK cells were defined (Figure S3B). The NK1 population exhibited the highest CD56 expression, while NK2 showed medium to low expression. Subsequently, FlowSOM and Cluster Explorer were applied to three different samples to identify clusters and access changes in phenotypes and CD99 ligand expression across NK cells, CD3^+^ cells, and monocytes.

**Figure 3:**
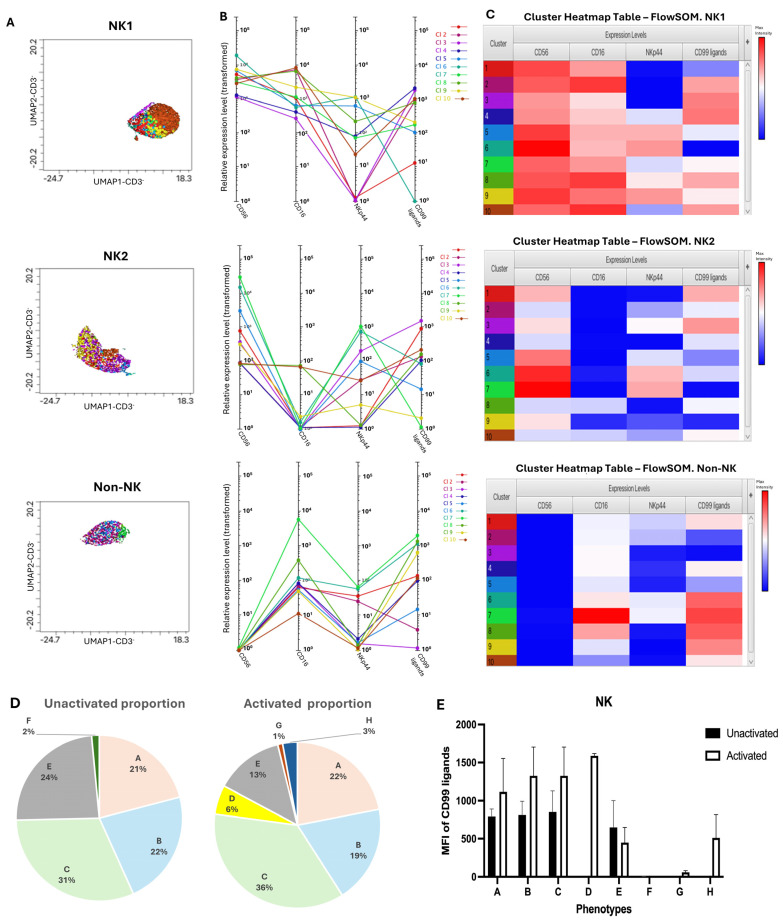
Phenotypic clustering of NK and non-NK populations using FlowSOM and Cluster Explorer. (**A**–**C**) Figures generated from Cluster Explorer to visualize and interpret clusters revealed with FlowSOM. Different colors in the heatmap represent different clusters identified by FlowSOM. (**A**) The overlaid dimensionality reduction plots show the position of each cluster from NK1, NK2, and non-NK populations with different colors. (**B**) The profile charts display the expression patterns of activation markers and CD99 ligands across 10 clusters from each population and (**C**) heatmaps demonstrating the relative intensity of activation markers and CD99 ligands. (**D**) The two pie charts show the average proportion of NK phenotypes (A–H) in unactivated and activated conditions. (**E**) The bar graph showed the MFI of CD99 ligand expression across eight NK phenotypes (A–H) (n = 3). Statistical analysis was performed using a paired *t*-test. Data are presented as mean ± SEM.

To investigate the cellular distribution of CD99 ligands among various NK cell phenotypes, NK (NK1 and NK2) and non-NK populations were further analyzed with flowSOM and Cluster Explorer using two distinct staining panels with different antibody combinations, enabling the identification of distinct NK cell phenotypes. The first panel defined phenotypes by the expression levels of CD56, CD16, and NKp44, while the second used CD56, CD69, and CD137. Using the first staining panel, FlowSOM and Cluster Explorer analysis initially identified a total of 10 clusters across all samples (Figure 3A–C). From these, only clusters with a high proportion (>1%) and consistent presence across all donors were selected. These selected clusters were subsequently grouped into eight phenotypes (A–H), which were defined based on the levels of CD56, CD16, and NKp44 (Table 1). The average relative intensity was measured by the MFI of CD99 ligands expressed on these phenotypes. For CD56DimCD16^+^ phenotypes (A–C), NKp44 was detected at different levels. All of these phenotypes can be seen in both resting and activated conditions, with a slight increase in proportion after activation. Additionally, the MFI of CD99 ligands increased upon activation; however, no statistically significant difference was observed before and after activation (Figure 3D,E). Among CD56DimCD16^−^ phenotypes (D–E), group D (NKp44++) was found only under activated conditions, exhibiting a high level of CD99 ligand expression. Group E, which shows low expressions of NKp44, was present in both conditions and displayed minimal changes in CD99 ligand expression. The CD56 Bright phenotypes were categorized into groups F–H based on the expression levels of CD16 and NKp44. These CD56 Bright subsets exhibited low expression of the CD99 ligands, except for group H. When comparing CD56 Bright and Dim populations, the expression level of CD99 ligands was lower in the CD56 Bright subsets. Overall, IL-2 activation resulted in the increased expression of activation markers and a higher level of CD99 ligands (Figure 3).

In non-NK cells, only a single phenotype, defined as CD56-CD16-NKp44- and labeled as group A, showed a proportion greater than 1% across all three donors. This phenotype was observed before and after IL-2 activation, exhibiting the expression of CD99 ligands. Following activation, the proportion of this phenotype increased, but the expression level of CD99 ligands decreased (Table 2).

**Figure 4:**
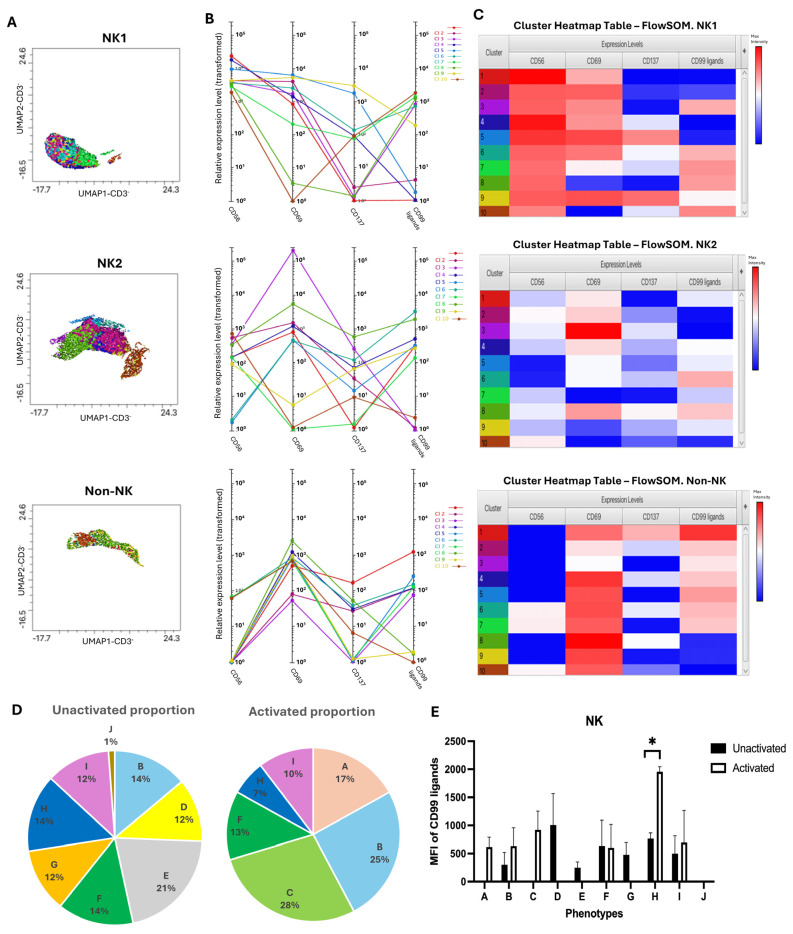
The identification of NK and non-NK clusters using FlowSOM and Cluster Explorer. (**A**–**C**) Figures generated from Cluster Explorer to visualize and interpret clusters revealed with FlowSOM. Different colors in the heatmap represent different clusters identified by FlowSOM. (**A**) The overlaid dimensionality reduction plot, (**B**) profile chart, and (**C**) cluster heatmap table show the position of clusters, MFI of each marker, and the expression level of CD99 ligands. The 10 clusters identified from each group were classified based on expression levels of CD69 and CD137. (**D**) Two pie charts demonstrate the average proportion of each phenotype under unactivated and activated conditions. Only phenotypes with a frequency greater than 1% and detected in all donors were included in the analysis. (**E**) The bar graph showed the average MFI of CD99 ligands expressed on 10 different phenotypes (A–J). Statistical analysis was performed by a paired *t*-test. Data are presented as mean ± SEM. * *p* ≤ 0.05.

Further, the second staining panel was employed, in which NK cells were identified and subdivided into NK1 and NK2 based on the expression of CD56, CD69, and CD137. FlowSOM and Cluster Explorer identified 10 distinct clusters (Figure 4). Although the initial FlowSOM analysis was set to generate 10 clusters, subsequent phenotypic classification yielded 11 distinct phenotypes. However, only 10 phenotypes are presented in Figure 4D,E, as one phenotype (group K) was detected in only one donor with a proportion of >1% and, therefore, excluded from comparative analysis. As shown in Table 3, phenotypes of CD56 Dim showed notable changes upon activation, particularly in relation to CD69 expression. Groups A–C, characterized by high CD69 expression, showed increased proportions and elevated CD99 ligand MFI compared to the unactivated state. These phenotypes represent activated CD56 Dim NK cells with varying CD137 expression. Groups D and E, with medium expression of CD69, expressed either high or low levels of CD99 ligands before activation; however, these subsets were absent after activation, suggesting a possible transition into other activated phenotypes. The other subsets of CD56 Dim, which expressed low or no CD69 (F–I), showed medium expression levels of CD99 ligands before activation. Although their proportions remained relatively unchanged, group G disappeared, and subsets H and I displayed increased CD99 ligand expression after activation. These data suggest that activation with IL-2 enhanced the expression of CD69 on CD56 Dim NK cells, accompanied by upregulated CD99 ligand expression, (Figure 4). In contrast, groups J and K, which represent CD56 Bright NK cell phenotypes, were very rare or detected in only one donor. Group J (phenotype showing CD56BCD69-CD137-) constituted only 1% of the unactivated population and was not observed following activation. The CD99 ligand expression in this subset was negligible (MFI = 2.03). Group K (CD56BCD69++CD137-) appeared at a low frequency (1.2%) in the activated state in only one donor and showed minimal CD99 ligand expression even after activation (MFI = 1.1). Although other donors also exhibited CD56 Bright phenotypes, their proportions were consistently lower than 1% and were therefore excluded from the analysis (Table 3).

In this staining condition, although 10 different non-NK cell phenotypes were detected, each phenotype was present at a very low proportion (<1%). Moreover, some phenotypes were only observed in a single donor and were not consistent across all donors, making it difficult to identify and define the phenotypes.

To summarize, CD99 ligands were expressed on NK cells both before and after activation. However, activation leads to alterations in NK cell phenotypes, resulting in a higher expression of activation markers and an increased average proportion. As the proportion of these phenotypes rises, the MFI of CD99 ligands on these phenotypes also increases. However, some phenotypes with upregulated activating receptors exhibited higher levels of ligand expression, even though their proportions did not increase significantly. Furthermore, compared to CD56 Dim and CD56 Bright NK cells, an increased distribution of CD99 ligands was observed on the CD56 Dim subsets in both unactivated and IL-2-activated conditions.

**Figure 5:**
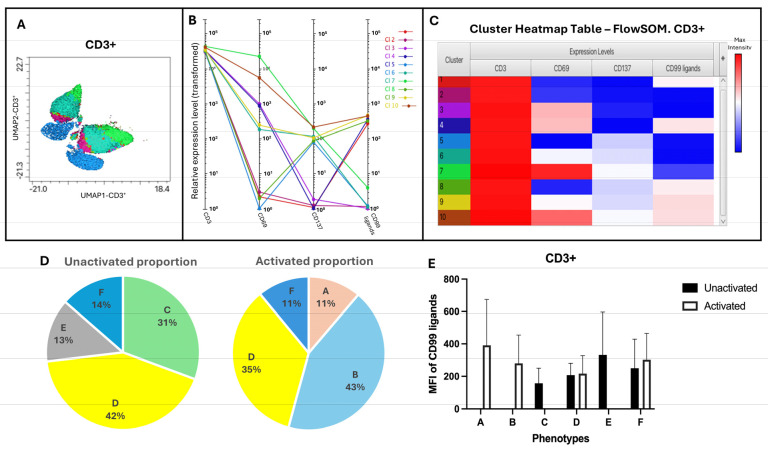
Phenotypic clustering of CD3^+^ cells using FlowSOM and Cluster Explorer. (**A**–**C**) Figures generated from Cluster Explorer to visualize and interpret clusters identified by FlowSOM. Different colors in the heatmap represent different clusters identified by FlowSOM. (**A**) The overlaid dimensionality reduction plots, (**B**) the profile charts, and (**C**) heatmaps demonstrating the relative intensity of activating markers and CD99 ligands for 10 different clusters. CD3^+^ cells were further categorized into six different phenotypes based on the expression level of CD69 and CD137. (**D**) The pie charts demonstrate the average proportion of each phenotype. (**E**) The bar graph shows the average MFI of CD99 ligands expressed on six different phenotypes (A–F) (n = 3). Statistical analysis was performed using a paired *t*-test. Data are presented as mean ± SEM.

CD69 and CD137, markers expressed on CD3^+^ cells, were used to determine different phenotypes of CD3^+^ population cells to examine their differences in CD99 ligand expression. The use of FlowSOM and Cluster Explorer determined 10 clusters with different expression levels of CD69 and CD137 (Figure 5A–C). Of the 10 different clusters generated by FlowSOM and Cluster Explorer, low-proportion clusters (<1%) were excluded, and the remainders were classified into six phenotypes (A–F) according to the expression level of CD69 and CD137 activation markers (Table 4). The average proportion of each phenotype before and after stimulation with IL-2, and the average MFI of CD99 ligands obtained from the heatmap were calculated for three different samples. As shown in Table 4, activated T cell phenotypes A and B were not seen in unactivated conditions but appeared in activated states according to the expression of activation markers CD69 and CD137. These phenotypes showed higher proportions and higher fluorescence intensity of CD99 ligands compared to other phenotypes. Phenotype D shows no observable difference in either ligand expression or population proportion before and after stimulation. In phenotype F, although the proportion of the population shows little change, the MFI increases after stimulation. Some phenotypes are activated before stimulation with IL-2. These are phenotypes C and E, which showed a high expression of activating receptors CD137 but not CD69. These phenotypes were observed only before activation, but the effect of activation with IL-2 changed the expression of markers, and these phenotypes disappeared.

These data demonstrated that, in the CD3^+^ population, the transition of phenotypes can be observed after activation with IL-2. The phenotypes C and E, which expressed low and medium levels of CD69 and CD137, shifted to high-level-expression phenotypes A and B. Moreover, these altered phenotypes showed a higher MFI of CD99 ligands along with high proportions (Figure 5D,E).

**Figure 6:**
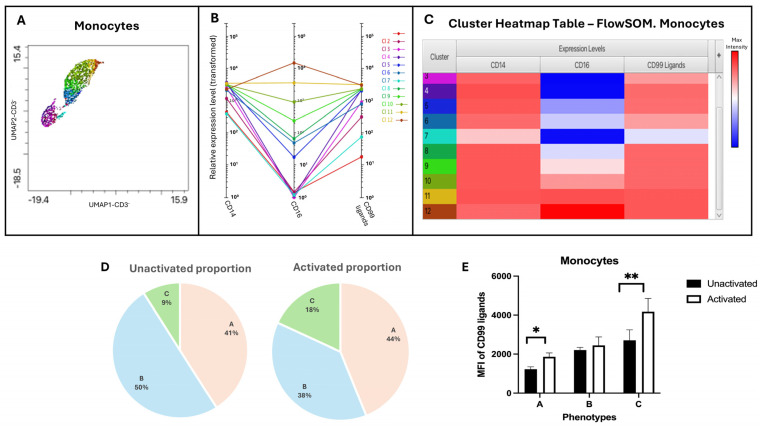
Identification and characterization of distinct monocyte subsets using FlowSOM and Cluster Explorer. (**A**–**C**) Figures generated from Cluster Explorer to visualize and interpret clusters revealed with FlowSOM. Different colors in the heatmap represent different clusters identified by FlowSOM. (**A**) The overlaid dimensionality reduction plots demonstrating the 12 populations. (**B**,**C**) The profile charts and the cluster heatmap tables showing the intensity of each marker and the expression level of CD99 ligands. These clusters were categorized based on varying levels of CD14 and CD16 expression, resulting in three different phenotypes of monocytes. (**D**) The pie charts illustrate the distribution of each phenotype. (**E**) The bar graph presents the MFI of CD99 ligands expressed on three distinct phenotypes (A–C), with a sample size of n = 3. Statistical analysis was performed with a paired *t*-test. Data are presented as mean ± SEM. * *p* ≤ 0.05, ** *p* ≤ 0.01.

Different monocyte phenotypes were identified using FlowSOM and Cluster Explorer, which revealed 12 clusters (Figure 6A–C). These clusters were further categorized into three major subsets of monocytes according to the expression of CD14 and CD16, classical (CD14++CD16−), intermediate (CD14++CD16+), and non-classical (CD14+CD16++). CD99 ligand expressions across these subsets of monocytes were examined from the heatmap generated by Cluster Explorer. All subsets of monocytes showed a high level of CD99 ligands, with the highest level found in non-classical phenotypes (Table 5 and Figure 6D,E). The expression level of CD99 ligands appeared to be slightly higher after activation with IL-2. In terms of population distribution, the proportion of the classical monocytes, CD14++CD16−, remained unchanged before and after activation. However, activation with IL-2 resulted in the upregulation of CD16, and there was a transition of group B (CD14++CD16+) to group C (CD14+CD16++) following activation (Table 5). This was reflected by a decrease in the proportion of intermediate monocytes (group B) and a two-fold increase in the non-classical subset (group C) upon activation. Consequently, a higher MFI of CD99 ligands in non-classical monocytes was observed along with an increased proportion (Figure 6D,E).

## 4. Discussion

The interaction between ligands and leukocyte surface receptors plays a pivotal role in the mechanisms of immunoregulation. The transmembrane protein, CD99, has been shown to be involved in many biological processes [1,2]. Recently, under optimized conditions, we could produce large quantities of recombinant CD99 proteins (named CD99HIgG) [3]. This enables us to investigate the presence of CD99 ligands and their involvement in immune regulation. Using the produced CD99HIgG, we demonstrated the presence of CD99 ligands on the surface of various leukocytes including monocytes, NK cells, and dendritic cells and their involvement in the regulation of cytokine production [4].

In this study, we further examined the presence of CD99 ligands on IL-2-activated NK cells, CD3^+^ cells, and monocytes. IL-2 regulates the functions of leukocytes by binding to IL-2 receptors [10] and induces cell activation. IL-2 is a central cytokine that directly stimulates T cells and NK cells, which express IL-2 receptors. These activated lymphocytes produce cytokines such as IFN-γ, which can indirectly activate monocytes [11,12]. By using multicolor immunofluorescent staining and flow cytometry, the conventional flow cytometric gating allows for the analysis of predefined subsets. However, it relies on 2D plots and user-defined cutoffs, and so it is limited by human bias and unable to visualize relationships among multiple markers simultaneously. To overcome these limitations, high-dimensional analysis tools UMAP, FlowSOM, and Cluster Explorer were employed. These methods can analyze the full spectrum of marker expression simultaneously. The limitations of user bias and the inability to visualize rare populations, which might be overlooked by conventional gating, could be overcome. We have combined conventional and high-dimensional analysis to carry out a comprehensive assessment of CD99 ligand distribution across different immune subsets.

Conventional analysis revealed the CD99 ligand expression in NK cells and monocytes and showed that it further increased following IL-2 stimulation. In CD3^+^ cells, however, there was no expression of CD99 ligands in the unactivated state. Activation with IL-2 induced a slight increase in the CD99 ligand level. There is currently no information regarding the cellular distribution of these ligands among various phenotypes of PBMCs. High-dimensional analysis showed the alteration of phenotypes in the PBMC population upon IL-2 stimulation. CD99 ligands are dynamically distributed across these phenotypes, and a higher expression of CD99 ligands is correlated with the upregulation of activation markers. However, there was no statistical difference in expression among most phenotypes due to inter-individual variability and differences in baseline expression levels across donors, and some phenotypes appeared only after activation.

NK cell phenotypes were defined based on CD56, CD16, NKp44, CD69, and CD137 expression. CD16 is normally expressed on NK cells and increases with IL-2 activation, while NKp44 is only expressed on activated NK cells but not on resting cells [13]. Therefore, it is a useful marker for identifying activated NK cells. CD69, an early activation marker, appears within two hours after stimulation, peaks after 18–24 h, and then declines. The expression of CD137 increases after 5 h of activation, peaks by 24 h, and gradually declines within 48–72 h [14]. Using a clustering algorithm, new phenotypes expressing a higher level of NKp44 were observed after activation. Upon stimulation, the proportion of the activated phenotypes showed an upward trend accompanied by elevated CD99 ligand expression. In addition, CD99 ligand expression could be observed on CD16-negative NK phenotypes, indicating that our observations of ligand expression are not due to Fc receptor-mediated binding. Phenotypes defined by CD69 and CD137 showed that certain unactivated phenotypes have transitioned to activated states, and these newly formed subsets exhibit an increased expression of CD99 ligands. These data suggest that the higher CD99 ligand level might be associated with the upregulation of activation markers.

Approximately 50% of CD3^+^ cells were activated with intermediate levels of CD137 but low CD69. Under standard culture conditions, PBMCs can release paracrine cytokines and growth factors [15] and regulate the expression of activation receptors even before the addition of IL-2. High-dimensional analysis showed low CD99 ligand levels across all CD3^+^ phenotypes; therefore, in conventional analysis, CD99 ligand expression appears to be absent. Activation with IL-2 upregulated activation markers caused spontaneously activated phenotypes to either disappear or switch to more activated subsets that displayed the highest CD99 ligand expression. Although CD99 ligand levels trended upward, they remained lower than NK cells and monocytes. Consistent with previous reports [8], our findings confirm that CD99 ligands are upregulated upon T cell activation and may regulate cytokine production.

Monocytes are heterogeneous and can be divided into classical (CD14++CD16−), intermediate (CD14++CD16+), and non-classical (CD14+CD16++) types [16]. In humans, classical monocytes comprise ∼85%, and the remaining ∼15% are intermediate and non-classical subsets [17]. Classical monocytes produce anti-inflammatory cytokines such as IL-10, while the intermediate monocytes produce pro- and anti-inflammatory cytokines (TNF-α and IL-10). The non-classical subset induces the production of pro-inflammatory cytokines including IL-6, IL-1β, and TNF-α [18,19]. Spontaneously activated phenotypes were detected, likely caused by cytokines such as TGF-β being released from PBMCs [20] or by activated platelets, both of which can convert CD14+CD16− monocytes into CD14+CD16+ ones [21]. Monocytes express the receptors for a variety of growth factors including the IL-2 receptor [22,23]. Upon IL-2 stimulation, CD16 expression increased, doubling the proportion of non-classical monocytes. All three subsets of monocytes expressed CD99 ligands, with the highest levels observed in the non-classical monocytes, and expression nearly doubled after stimulation. These non-classical monocytes show a high expression of CD16 and FcγR, and the recombinant CD99 protein is fused with the Fc part of HIgG. To investigate whether the highest CD99 ligand expression on this subset is due to the Fc-mediated binding, ligand expression was compared between conditions of no protein, control CD147Rg, and CD99HIgG. Only CD99HIgG showed strong binding, confirming the specific interaction of CD99 and its ligands (Table S1). CD99 ligands on monocytes are known to mediate pro-inflammatory cytokine production [8]. Our high-dimensional analysis revealed the increased CD99 ligand expression on non-classical monocytes, a subset responsible for producing pro-inflammatory cytokines. This finding suggests that the enhanced expression of CD99 ligands on this subset reinforces their pro-inflammatory role.

Although our study demonstrates the distribution of CD99 ligands, their exact identity remains unknown. Further studies are still needed to determine which ligands are functionally significant. Several potential binding partners have been suggested in previous studies. CD99 itself can act as a ligand through homophilic interactions and plays essential roles in the diapedesis of leukocytes [7] and the homotypic aggregation of CD4+CD8+ thymocytes [24]. In addition, melanocytes communicate with various stromal and immune cell types primarily via CD99-CD99 binding, while interactions with macrophages may involve CD99-CD99L2 engagement [25]. In mice, interactions between CD99 and its ligand, PILR, regulate thymocyte apoptosis [6]. However, PILR binds to mouse CD99 but not human CD99 [26]. GDF6 has also been reported to bind to CD99 and to regulate Src signaling in Ewing sarcoma [5], although it is not expressed in immune cells [27]. Taken together, these studies suggest that both homophilic and heterophilic CD99 interactions may underlie the dynamic changes in ligand expression observed on PBMCs and influence immune activation.

## 5. Conclusions

In conclusion, we demonstrated the presence of CD99 ligands on resting and IL-2-activated NK cells and monocytes. In CD3^+^ cells, CD99 ligand expression was very low at baseline but increased upon activation. CD99 ligands were distributed across different phenotypes of PBMCs and were upregulated upon IL-2 stimulation. Therefore, we hypothesize that the amplification of CD99 ligand expression is associated with an increased level of activating markers. While further studies are still needed to clarify the roles of CD99 and its ligands in immune responses and disease processes, our study provides initial insights into the regulation of CD99 ligands on IL-2-activated PBMCs and establishes a foundation for further investigations.

## Figures and Tables

**Table 1:** High-dimensional analysis of NK cells showing eight different phenotypes depending on expression level of CD56, CD16, and NKp44. Average MFI of CD99 ligand expression and proportion of phenotypes (n = 3). CD56D; CD56 Dim and CD56B; and CD56 Bright.

Group	Phenotype	Unactivated Proportion	Activated Proportion	MFI of CD99 Ligands (Unactivated)	MFI of CD99 Ligands (Activated)
A	CD56D CD16+ NKp44++	21%	22%	792.75	1115.3
B	CD56D CD16+ NKp44+	22%	19%	811.341667	1326.9
C	CD56D CD16+ NKp44−	31%	36%	851.183333	1278.7
D	CD56D CD16− NKp44++		6%		1587.9
E	CD56D CD16− NKp44+	24%	13%	649.4875	450.1
F	CD56B CD16− NKp44++	2%		4.3367	
G	CD56B CD16− NKp44+++		1%		59.8
H	CD56B CD16+ NKp44+++		3%		511.6

**Table 2:** High-dimensional analysis of non-NK depending on expression level of CD56, CD16, and NKp44. Average MFI of CD99 ligand expression and proportion of phenotype (n = 3).

Group	Phenotype	Unactivated Proportion	Activated Proportion	Average MFI of CD99 Ligands (Unactivated)	Average MFI of CD99 Ligands (Activated)
A	CD56Neg CD16− NKp44−	14%	24%	530.15	263.5

**Table 3:** High-dimensional analysis of NK showing eleven different phenotypes depending on expression level of CD56, CD69, and CD137. Average MFI of CD99 ligand expression and proportion of phenotypes (n = 3). CD56D; CD56 Dim, CD56B; CD56 Bright; and * *p* ≤ 0.05.

Group	Phenotype	Unactivated Proportion	Activated Proportion	MFI of CD99 Ligands (Unactivated)	MFI of CD99 Ligands (Activated)
A	CD56D CD69+++ CD137++		17%		618.13
B	CD56D CD69+++ CD137+	14%	25%	301.91	634.61
C	CD56D CD69+++ CD137−		28%		920.7
D	CD56D CD69++ CD137++	12%		1006.97	
E	CD56D CD69++ CD137−	21%		247.63	
F	CD56D CD69+ CD137+	14%	13%	637.06	603.8
G	CD56D CD69+ CD137−	12%		477.32	
H	CD56D CD69− CD137+	14%	7%	614.40 *	1955.85 *
I	CD56D CD69− CD137−	12%	10%	499.57	694.05
J	CD56B CD69− CD137−	1%		2.03	
K	CD56B CD69++ CD137−		1.2% (only one donor)		1.1 (only one donor)

**Table 4:** High-dimensional analysis of the CD3^+^ population showing eight different phenotypes. Average MFI of CD99 ligand expression and proportion of phenotypes (n = 3).

Group	Phenotype	Unactivated Proportion	Activated Proportion	MFI of CD99 Ligands (Unactivated)	MFI of CD99 Ligands (Activated)
A	CD3+ CD69++ CD137+++		11%		390.52
B	CD3+ CD69+ CD137+++		43%		279.53
C	CD3+ CD69+ CD137++	31%		151.27	
D	CD3+ CD69+ CD137-	42%	35%	207.93	216.25
E	CD3+ CD69− CD137++	13%		331.45	
F	CD3+ CD69− CD137−	14%	11%	154.85	302.08

**Table 5:** High-dimensional analysis of monocytes showing three different phenotypes. Average MFI of CD99 ligand expression and proportion of phenotypes (n = 3). * *p* ≤ 0.05, ** *p* ≤ 0.01.

Group	Phenotype	Unactivated Proportion	Activated Proportion	MFI of CD99 Ligands (Unactivated)	MFI of CD99 Ligands (Activated)
A	CD14++CD16−	41%	44%	1232.4 *	1863.5 *
B	CD14++CD16+	50%	38%	2210.3	2441.8
C	CD14+CD16++	9%	18%	2702.6 **	4174.8 **

## Data Availability

The original contributions presented in this study are included in the article/Supplementary Materials. Further inquiries can be directed to the corresponding author.

## Supplementary Materials

The following supporting information can be downloaded at: https://www.mdpi.com/article/10.3390/biology15010086/s1, Figure S1. Gating strategy for NK cells: SSC-H and SSC-A are used to gate singlets followed by plotting against FSC-A and SSC-A for the population of PBMCs. This gating was plotted against CD3-PECF594 and CD14-FITC, and then the CD3^−^CD14^−^ population was gated. After that, CD3^−^CD56^+^ NK cells were determined. The expression of CD99 ligands on NK cells was determined by using Streptavidin-PE. Figure S2. Gating strategy for CD3^+^ and monocytes; SSC-H and SSC-A were used to gate singlets followed by plotting against FSC-A and SSC-A for the population of PBMCs. This gating was plotted against CD3-PECF594 and CD14-FITC, and then CD3^+^ and CD14^+^ (monocytes) populations were gated. The expression of CD99 ligands on these two cell populations was determined by using Streptavidin-PE. Figure S3. Gating strategy and identification of CD3^−^ cell subsets using UMAP. (A) FSC-A and FSC-H were applied to determine singlets, which were further plotted between FSC-A and SSC-A to gate PBMC. CD3^+^ and CD3^−^ populations were gated by using SSC-A and CD3. After that, the CD3^−^ population was further divided into monocyte, NK, and non-NK cells using UMAP according to the expression of CD14, CD56, CD16, and NKp44. (B) The heatmaps of CD14, CD56, CD16, and NKp44 are shown. NK (NK1, NK2) and non-NK subsets were identified based on marker combinations. The color scale represents marker expression levels from low (blue) to high (yellow). Table S1. Representative donor demonstrating the comparison of ligand expression and proportion of non-classical monocyte subset between conditions of CD99HIgG, control CD147Rg, and no protein by high-dimensional analysis. Table S2. List of reagents used in the study.

## Abbreviations

The following abbreviations are used in this manuscript:

NK cell	Natural killer cell
UMAP	Uniform manifold approximation and projection
FlowSOM	Flow cytometry self-organizing map
IL-2	Interleukin 2
PBMC	Peripheral blood mononuclear cell
