# Supplementary material for: Phenotypic Profiling and Activation-Associated Expression of CD99 Ligands on Human Leukocytes
Source: Biology (Basel). 2025 Dec 31;15(1):86. doi: 10.3390/biology15010086 (PMC12785092; doi:10.3390/biology15010086)
Supplement: Supplementary file 1 [file biology-15-00086-s001.zip › biology-3958138-supplementary.pdf]

biology

MDPI

Supplementary Materials:

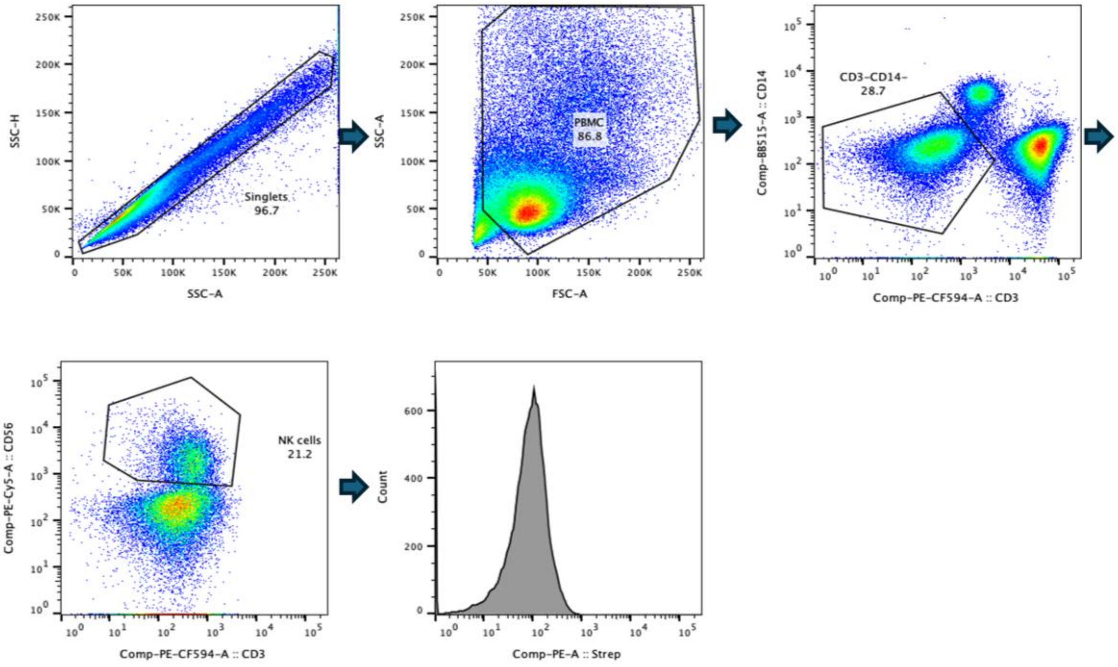

Figure S1. Gating strategy for NK cells: SSC-H and SSC-A are used to gate singlets followed by plotting against FSC-A and SSC-A for the population of PBMCs. This gating was plotted against CD3-PECF594 and CD14-FITC and then the $CD3^-CD14^-$ population was gated. After that, $CD3^-CD56^+$ NK cells were determined. The expression of CD99 ligands on NK cells was determined by using Streptavidin-PE.

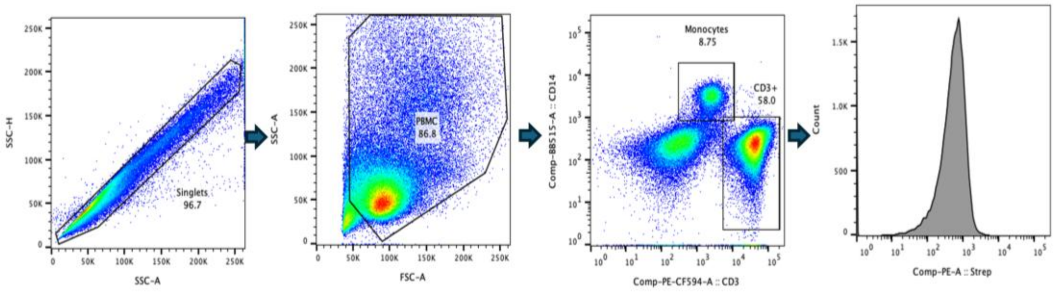

Figure S2. Gating strategy for CD3+ and monocytes, SSC-H and SSC-A were used to gate singlets followed by plotting against FSC-A and SSC-A for the population of PBMCs. This gating was plotted against CD3-PECF594 and CD14-FITC and then $CD3^+$ and $CD14^+$ (monocytes) populations were gated. The expression of CD99 ligands on these two cell populations was determined by using Streptavidin-PE.

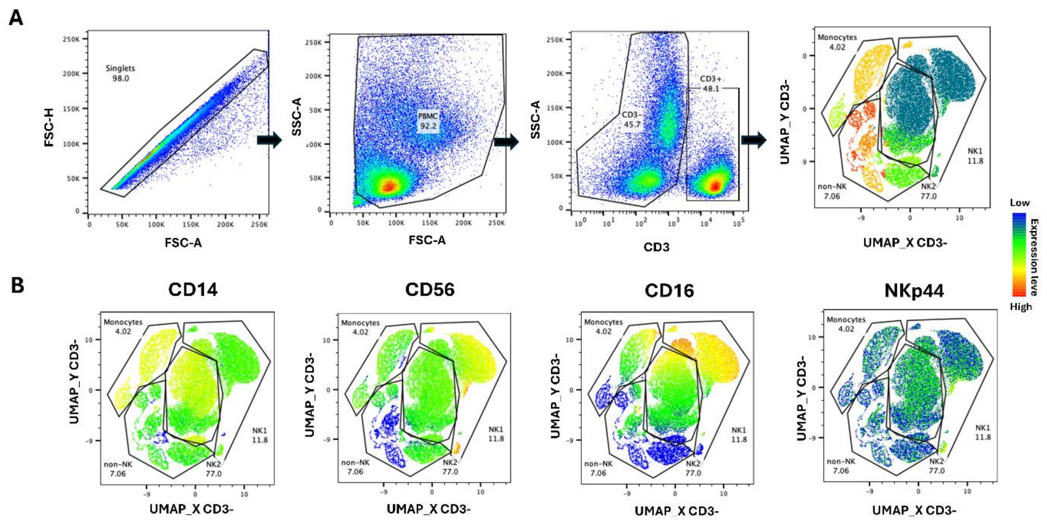

Figure S3. Gating strategy and identification of CD3⁻ cell subsets using UMAP. A) FSC-A and FSC-H were applied to determine singlets, which were further plotted between FSC-A and SSC-A to gate PBMC. $CD3^+$ and $CD3^-$ populations were gated by using SSC-A and CD3. After that, the $CD3^-$ population was further divided into monocyte, NK and non-NK cells using UMAP according to the expression of CD14, CD56, CD16, and NKp44. B) The heatmap of CD14, CD56, CD16 and NKp44 is shown. NK (NK1, NK2) and non-NK subsets were identified based on marker combinations. The color scale represents marker expression levels from low (blue) to high (yellow).

Table S1. Representative donor demonstrating the comparison of ligand expression and proportion of non-classical monocyte subset between conditions of CD99HIgG, control CD147Rg, and no protein by high-dimensional analysis

Conditions	Phenotype	Unactivated proportion	Activated proportion	MFI of CD99 ligands (unactivated)	MFI of CD99 ligands (activated)
CD99HIgG	CD14+CD16++	29.08	38.15	3821	6009
CD147Rg		28.49	24.6	1924	2270
No protein		8.7	21.07	168	325

Table S2. List of reagents used in the study

**Reagent**	**Brand**	**Category Number**	**Clone**
**Antibodies**			
PECF594 anti-human CD3 antibody	BD Biosciences	562280	UCHT1
PECy5 anti-human CD56 (NCAM) antibody	BioLegend	318308	HCD56

https://doi.org/10.3390/biology15010086

FITC anti-human CD14 antibody	BioLegend	301804	M5E2
BV711 anti-human CD16 antibody	BioLegend	302044	3G8
BV421 anti-human CD69 antibody	BioLegend	310930	FN50
PECy7 anti-human CD336 (NKp44) antibody	BioLegend	325116	P44-8
PECy7 anti-human CD137 antibody	BioLegend	309818	4B4-1
PE Streptavidin	BioLegend	405204	n/a
PECy7 Mouse IgG1 antibody	BioLegend	400126	MOPC-21
BV421 Mouse IgG1 antibody	BioLegend	400158	MOPC-21
**Chemicals, Cytokine and recombinant proteins**			
EZ-Link Sulfo-NHS-LC-Biotin	Thermo SCIENTIFIC	21335	
DTSSP (3,3′-dithiobis sulfosuccinimidylpropionate)	Thermo SCIENTIFIC	21578	
Human IL-2	Immunotools	200-02	
Human TruStain FcX™	BioLegend	422302	

Disclaimer/Publisher's Note: The statements, opinions and data contained in all publications are solely those of the individual author(s) and contributor(s) and not of MDPI and/or the editor(s). MDPI and/or the editor(s) disclaim responsibility for any injury to people or property resulting from any ideas, methods, instructions or products referred to in the content.

https://doi.org/10.3390/biology15010086
